# Social Defeat: Impact on Fear Extinction and Amygdala-Prefrontal Cortical Theta Synchrony in 5-HTT Deficient Mice

**DOI:** 10.1371/journal.pone.0022600

**Published:** 2011-07-27

**Authors:** Venu Narayanan, Rebecca S. Heiming, Friederike Jansen, Jörg Lesting, Norbert Sachser, Hans-Christian Pape, Thomas Seidenbecher

**Affiliations:** 1 Institute of Physiology I, Westfälische Wilhelms-University, Münster, Germany; 2 Department of Behavioural Biology, Westfälische Wilhelms-University, Münster, Germany; 3 Otto Creutzfeldt Center for Cognitive and Behavioral Neuroscience, Westfälische Wilhelms-University, Münster, Germany; University of Muenster, Germany

## Abstract

Emotions, such as fear and anxiety, can be modulated by both environmental and genetic factors. One genetic factor is for example the genetically encoded variation of the serotonin transporter (5-HTT) expression. In this context, the 5-HTT plays a key role in the regulation of central 5-HT neurotransmission, which is critically involved in the physiological regulation of emotions including fear and anxiety. However, a systematic study which examines the combined influence of environmental and genetic factors on fear-related behavior and the underlying neurophysiological basis is missing. Therefore, in this study we used the 5-HTT-deficient mouse model for studying emotional dysregulation to evaluate consequences of genotype specific disruption of 5-HTT function and repeated social defeat for fear-related behaviors and corresponding neurophysiological activities in the lateral amygdala (LA) and infralimbic region of the medial prefrontal cortex (mPFC) in male 5-HTT wild-type (+/+), homo- (−/−) and heterozygous (+/−) mice. Naive males and experienced losers (generated in a resident-intruder paradigm) of all three genotypes, unilaterally equipped with recording electrodes in LA and mPFC, underwent a Pavlovian fear conditioning. Fear memory and extinction of conditioned fear was examined while recording neuronal activity simultaneously with fear-related behavior. Compared to naive 5-HTT+/+ and +/− mice, 5-HTT−/− mice showed impaired recall of extinction. In addition, 5-HTT−/− and +/− experienced losers showed delayed extinction learning and impaired recall of extinction. Impaired behavioral responses were accompanied by increased theta synchronization between the LA and mPFC during extinction learning in 5-HTT-/− and +/− losers. Furthermore, impaired extinction recall was accompanied with increased theta synchronization in 5-HTT−/− naive and in 5-HTT−/− and +/− loser mice. In conclusion, extinction learning and memory of conditioned fear can be modulated by both the 5-HTT gene activity and social experiences in adulthood, accompanied by corresponding alterations of the theta activity in the amygdala-prefrontal cortex network.

## Introduction

The neurotransmitter serotonin (5-hydroxytryptamine, 5-HT) plays, besides vegetative and neuroendocrine functions, a key role in the physiological regulation of behavior, such as aggression, and emotions, such as mood and fear [1], [2]. Furthermore, 5-HT is implicated in the pathophysiology of psychiatric disorders such as impulse control disorders, depression and anxiety disorders [3], [4]. In this context, the serotonin transporter (SERT, 5-HTT) is a key regulator of central serotonergic activity [5]. The 5-HTT is a cell membrane protein that regulates the serotonin signaling via reuptake (clearance) of 5-HT from the extracellular space [6] to be recycled into presynaptic vesicles where it becomes anew available for serotonergic transmitter release [7]–[9], and plays a major role in regulating extracellular serotonin volume transmission [10].

Several studies have shown that anxiety and anxiety disorders can be influenced by both environmental and genetic factors [5], [11], [12]. Concerning genetic factors, the genetically encoded variation of 5-HTT expression is a mediator for the susceptibility to anxiety disorders [12]. Due to the major role as a key regulator during serotonergic neurotransmission, 5-HTT represents an important molecular target which is involved in the treatment of neuropsychiatric disorders (e.g. depression, anxiety and post-traumatic stress disorders (PTSD)) [13]–[22]. In humans and monkeys, a polymorphism in the 5-HTT gene regulatory region [23] was identified, resulting in an allelic variation of the 5-HTT expression, and associated traits of negative emotionality including depression and anxiety [23]–[26] and risk for PTSD [27].

The generation of mice with targeted disruption of the 5-HTT gene allows to examine the consequences of a diminished (e.g. in heterozygous mice) or absent function (in homozygous mice) of the 5-HTT. The 5-HTT mutant mouse model offers the opportunity to investigate potential adaptive changes in the 5-HT system in response to increased extracellular and decreased intracellular 5-HT levels [28] as well as to altered 5-HT synthesis and turnover [29]. Indeed, these 5-HTT knockout mice show depression-like behaviors, increased anxiety-like behavior and exhibit a selective deficit in extinction recall of fear memory [1], [30]–[33]. Thus, 5-HTT knockout mice provide an excellent model to examine the role of 5-HTT in processes of conditioned, retrieved and extinguished fear [25], [34]–[36].

Synaptic circuits, involved in the processing of aversive signals and the expression of anxiety-related behavior, including amygdala, prefrontal cortex and hippocampus, are vulnerable to environmental influences [37], [38]. For example, a single social defeat by a conspecific in adulthood is related to increases in anxiety-like behavior; at the same time the functionality of the 5HT1A receptor is decreased [39]. A recent study with 5-HTT knockout mice showed that after repeated social defeat 5-HTT −/− mice showed the highest level of anxiety-like behavior, as compared to 5-HTT +/− and +/+ animals [32]. Much data implicate critical interactions between lateral amygdala (LA) and medial prefrontal cortex (mPFC) in the regulation of affective behaviors, particularly in relation to fear and anxiety. The LA is involved in forming and maintaining conditioned and unconditioned stimulus associations and the infralimbic (IL) mPFC plays an important role in fear extinction-related processes [40], [41].

Neuronal oscillations (e.g. theta activity) display an efficient mechanism to mediate a coordinated inter-relation between participating brain regions (for review see [42]). Electrophysiological data showed that projection neurons in the LA generate oscillatory activity at theta frequencies [43]–[45] during fear related emotional arousal. Furthermore, Seidenbecher et al. [46] showed increased synchrony between the LA and the CA1 region of the hippocampus during consolidation and reconsolidation of conditioned fear. In addition, there is evidence for conditioned fear-related theta activity in the mPFC [47], and hippocampal-mPFC theta synchrony increases with anxiety [48]. Using multiple site electrophysiological recordings in freely behaving mice, it has been shown that theta synchrony provides a means for inter-areal coordination and information transfer [49]. Additionally, a functional magnetic resonance imaging study in humans revealed an increased amygdala-prefrontal coupling dependent on a genetic variation (carriers of the SLC6A4 short allele, [50]).

Although the potential influence of a genetic variation in 5-HTT function on human anxiety and fear behavior has been corroborated in studies of 5-HTT knock-out mice [36] the underlying neurobiological correlates of this functional relation are unknown. Therefore, the present study was undertaken to investigate the consequences of genotype specific disruption of 5-HTT function and negative social experience in adulthood (repeated social defeat in the resident-intruder paradigm) for fear extinction and underlying neurophysiological mechanisms in a 5-HTT knock-out mouse model. We particularly focused on amygdala-prefrontal cortex circuits and related synchronized theta activities in view of their role in fear-related behaviors.

Here we hypothesized that, fear extinction, related neurophysiological activities in LA and IL of mPFC and directionality of theta synchrony are influenced by i) genotype (loss or diminished 5-HTT function), ii) loser experience and iii) genotype X social experience interaction.

## Results

### Impaired extinction recall of fear memory in 5-HTT^−/−^ mice

**Figure 1:**
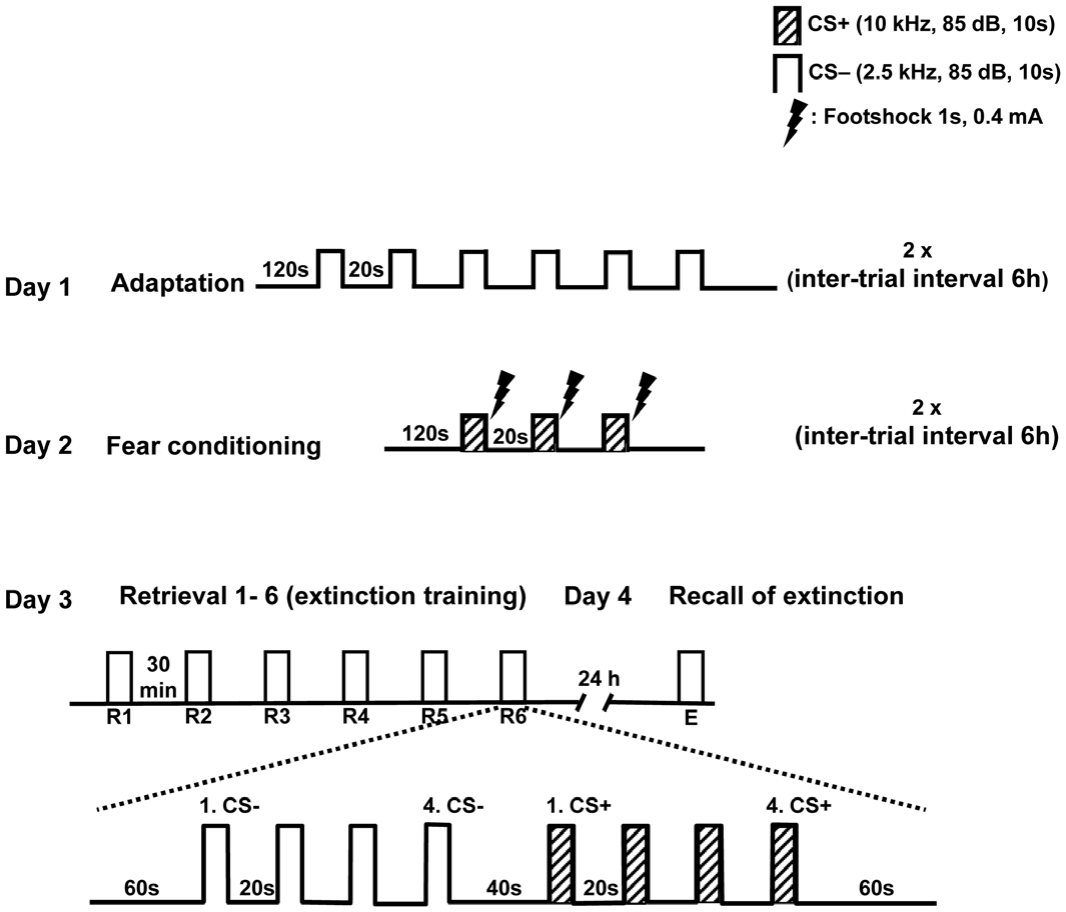
Schematic representation of experimental design. During adaptation, animals were exposed to six CS**^−^** only and the entire session was repeated six hours later (Day 1). Conditioning took place on the following day (Day 2): the CS**^+^** was presented three times, co-terminated with an electric footshock, during each conditioning session. Conditioned fear memory was tested on day 3 ( = extinction training); six consecutive extinction training sessions were carried out (R1 to R6), with 30 minutes intervals between sessions. Extinction memory was recalled on day 4 in one session (E). All sessions on day 3 and 4 were identical, and contained four CS**^−^** and four CS**^+^** presentations. Stimulus parameters and intervals are marked.

Male 5-HTT knockout (**^−/−^**, n = 10; **^+/−^**, n = 11) and wild-type mice (n = 12) were fear conditioned with a Pavlovian fear conditioning paradigm. Freezing behavior was assessed during the retrieval/extinction sessions (R1-R6), with repetitive presentations of conditioned (CS^+^) and neutral (CS^−^) stimuli, and during recall of extinction (E) twenty-four hours later (method in detail see also Figure 1).

**Figure 2:**
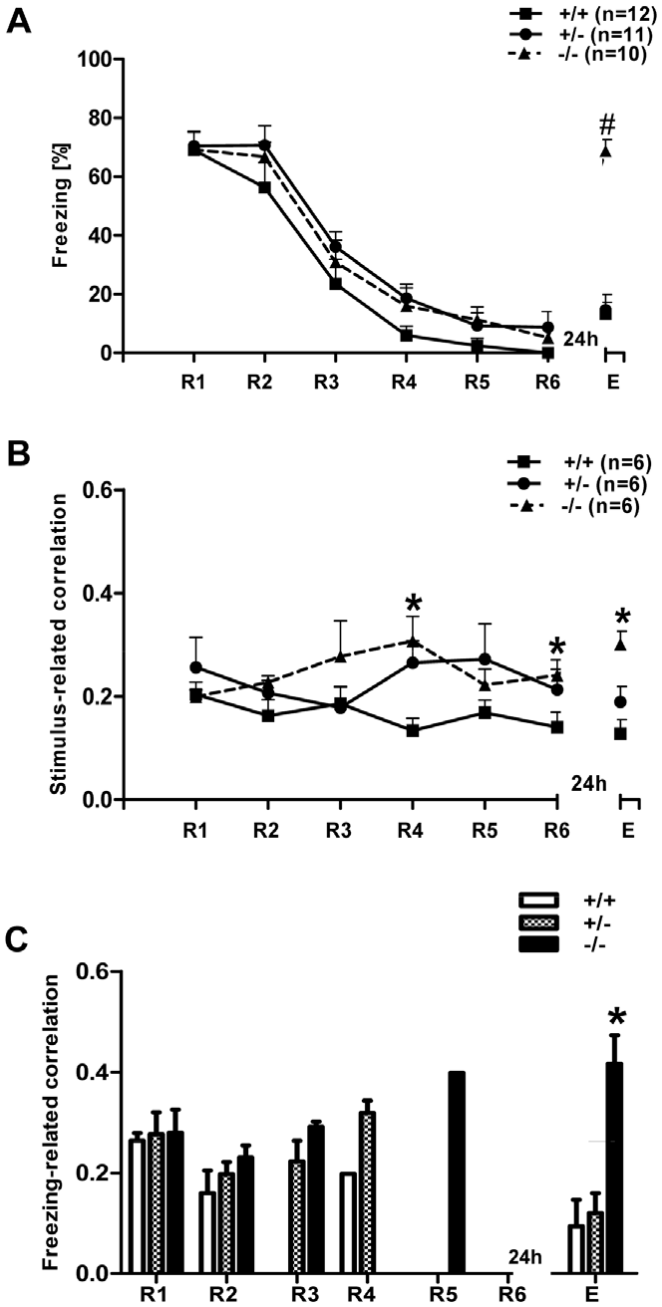
Freezing behavior and theta synchronization in naive 5-HTT mice at different stages of fear extinction. **A)** Freezing behavior during the first CS**^+^** across R1-R6 and E in all three genotypes. Freezing in all the three genotypes declined from R1-R6, indicating the standard extinction of fear memory. Twenty four hours later, when all the three genotypes were tested for fear extinction recall, 5-HTT**^−/−^** mice showed significant impaired extinction recall (E), represented by significantly higher levels of freezing compared to 5-HTT**^+/−^** and 5-HTT**^+/+^** mice (p<0.001). **B)** Stimulus-related theta correlation in 5-HTT mice at different stages of fear extinction. Averaged cross-correlation during the presentation of the first CS**^+^** between LA and IL of mPFC during extinction (R1-R6) and extinction recall (E) in 5-HTT naive mice is shown. When compared with 5-HTT**^+/+^**, 5-HTT**^−/−^** mice showed significantly increased cross-correlation during extinction training (note significant at R4 and R6; p<0.05). In 5-HTT**^−/−^** mice, the correlation values remained high across the extinction training and being significantly higher than for 5-HTT**^+/+^** mice during E. **C)** Freezing-related averaged cross-correlation during the first CS**^+^** between LA and IL of mPFC during extinction training (R1-R6) and extinction recall (E) in 5-HTT naive mice. Note, that the 5-HTT**^−/−^** mice showed significantly increased cross-correlation during extinction recall (p<0.05, compared to 5-HTT**^+/−^** and 5-HTT**^+/+−^** mice). Values are mean ± SEM; *p<0.05, #p<0.001.

During presentation of the CS+, all three genotypes showed a significant reduction of freezing behavior during retrieval and low freezing at the end of the extinction session, demonstrating successful extinction of conditioned fear memory. Twenty four hours later, when we tested for extinction recall, the 5-HTT^−/−^ mice displayed a pronounced level of freezing behavior compared to 5-HTT^+/+^ and ^+/−^ mice, demonstrating the disrupted ability of 5-HTT^−/−^ mice to retain the extinction of fear memory (Figure 2A). Repeated measures of one-way ANOVA with genotype (^+/+, +/−,^^−/−^) as the independent variable and session (R1-R6 and E) as dependent variable revealed a significant interaction between genotype and session (F (12, 18) = 3.85, p = 0.00003), as well as a significant main effect of both session (F (6, 180) = 55.99, p = 0.00001) and genotype (F (2, 30) = 4.09, p = 0.026). When compared with wild-type littermates, 5-HTT**^−/−^** and 5-HTT**^+/−^** exhibited the same freezing across R1-R6 (post hoc Bonferroni's test). All three genotypes showed no differences in fear acquisition and a reduction in freezing from R1 to R6 (significantly different from R3 onwards; repeated measures one-way ANOVA, F (6, 18) = 55.99, p = 0.00001; post hoc Tukey's test (same significant values in all three genotypes): R1 vs. R3, p<0.0001; R1 vs. R4, p<0.0001; R1 vs. R5, p<0.0001; R1 vs. R6, p<0.0001). However, 24 hours after extinction training, 5-HTT**^−/−^** mice exhibited a significant increase in freezing duration at extinction recall (E) compared to 5-HTT**^+/−^** (p<0.0001) and 5-HTT^+/+^ littermates (p<0.0001, post hoc Bonferroni's test).

The freezing behavior during the presentation of CS**^−^** revealed no significant differences between the genotypes during extinction learning and extinction recall (data not shown).

In summary, compared with 5-HTT^+/+^ and ^+/−^ mice, 5-HTT^−/−^ mice displayed a pronounced impairment of fear extinction recall.

### Synchronized theta activity during fear memory extinction in amygdala-prefrontal cortex network in 5-HTT knockout and wild-type mice

**Figure 3:**
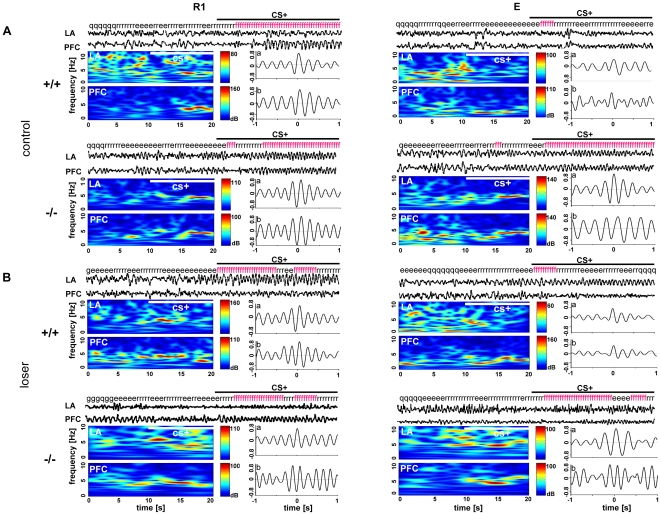
Theta synchronization in different stages of fear extinction in 5-HTT (^+/+^ and ^−/−^) (A) control and (B) socially defeated (loser) mice. Local field potentials (LFPs) from LA, and PFC were simultaneously recorded during early retrieval (R1) and extinction recall while monitoring the animals' behavior. The figure shows representative LFP traces (20 s) for each recording area from R1 and E during the first CS^+^ presentation for a control and a loser 5-HTT^+/+^ and ^−/−^ mouse, respectively. The behavior of the mice at the particular time is indicated above the traces (f, freezing; r, risk assessment; e, exploration; g, grooming; q, quiet), and wavelet transforms of the 20 s segments for each recording area are displayed underneath. The (a) stimulus- and (b) freezing-related cross-correlations are shown beneath the LFP traces, indicating theta synchronization. Independent of the genotype, both the control and socially defeated mice showed increased freezing behavior accompanied by high stimulus- and freezing-related theta synchrony at R1. Note, during recall of extinction (E), the 5-HTT^−/−^ control and loser mice showed increased freezing behavior and increased theta synchrony, whereas the 5-HTT^+/+^ control and loser mice showed low freezing behavior and low theta synchronization between LA and PFC.

In 5-HTT knockout (**^−/−^**, n = 6; **^+/−^**, n = 6) and wild-type mice (n = 6), recordings of local field potentials (LFPs) from LA and IL of mPFC were simultaneously conducted during CS**^+^** presentation in R1–R6 and E of cued fear (standard training) while monitoring the behavior. Since previous results demonstrated a change in theta frequency synchronization in the LA-CA1 [46] and in the LA-CA1-PFC network [46], [47] after fear conditioning during freezing behavior, stimulus-related and freezing-related correlations were separately calculated during the first CS^+^ presentation between the LA and mPFC (Figure 2B and C, see also Figure 3A).

Cross-correlation analyses of the LA and mPFC during retrieval (R1-R6) of fear memory in naive mice revealed highly synchronized theta activity at a frequency of ∼4 Hz in all three genotypes.

In detail, during the presentation of the conditioned stimulus (CS**^+^**), cross-correlograms showed a high level of synchronized theta activities between the LA and mPFC during extinction and extinction recall, as reported previously [47] (Figure 2B, see also Figure S1). A repeated measures of one-way ANOVA of LA-mPFC cue-related correlation values with genotype (^+/+, +/−, −/−^) as the independent variable and session (R1-R6 and E) as dependent variable revealed no significant interaction between genotype and session (F (12, 90) = 1.26, p = 0.259). However, there was a main effect of genotype (F (2, 15) = 5.56, p = 0.015), but not on session (F (6, 90) = 0.383, p = 0.89). When compared with 5-HTT**^+/+^** littermates, 5-HTT**^−/−^** exhibited significantly higher correlation values at R4, R6 and E (post hoc Bonferroni's test, p<0.05). The 5-HTT^+/−^ genotype displayed no difference in the theta synchrony compared with either 5-HTT^+/+^ and 5-HTT^−/−^ mice (post hoc Bonferroni's test).

In a next experimental step, correlated activity during freezing episodes was analyzed. Analysis was restricted to sessions R1, R2 and E, because here sufficient freezing behavior existed to obtain a cross-correlogram. In extinction training and recall, average cross-correlogram analysis of field potential waveforms recorded in LA and mPFC during defined freezing episodes revealed a high level of synchronized activities between both brain regions in all three genotypes (Figure 2C).

A repeated measures of one-way ANOVA of LA-mPFC freezing-related correlation values with genotype (^+/+, +/−, −/−^) as the independent variable and session (R1, R2 and E) as dependent variable revealed no significant interaction between genotype and session (F (2, 14) = 0.03, p = 0.969), but a main effect of genotype (F (2, 8) = 7.79, p = 0.013). Post hoc Bonferroni's test showed that freezing-related correlations in HTT**^−/−^** mice were significantly higher than in HTT**^+/+^** and HTT**^+/−^** at E (p<0.05). This result indicated that the 5-HTT**^−/−^** mice showed highly synchronized theta activities during freezing episodes at E. Cross-correlogram analyses during presentation of CS**^−^**, analyzed by one-way ANOVA for repeated measures, revealed no differences between the genotypes during extinction and extinction recall (data not shown).

In summary, 5-HTT**^−/−^** mice showed high amygdala-prefrontal cortex theta synchrony during late phases of fear extinction learning and recall of extinction, particularly during episodes of freezing behavior.

### Impaired fear extinction in 5-HTT ^+/+, +/−, −/−^ socially defeated (loser) mice

Here, after fear conditioning, freezing behavior during retrieval and extinction recall in socially defeated 5-HTT^+/+, +/−, −/−^ mice (generated by means of the resident-intruder paradigm, for details see Jansen et al. [32] and method part) was assessed.

**Figure 4:**
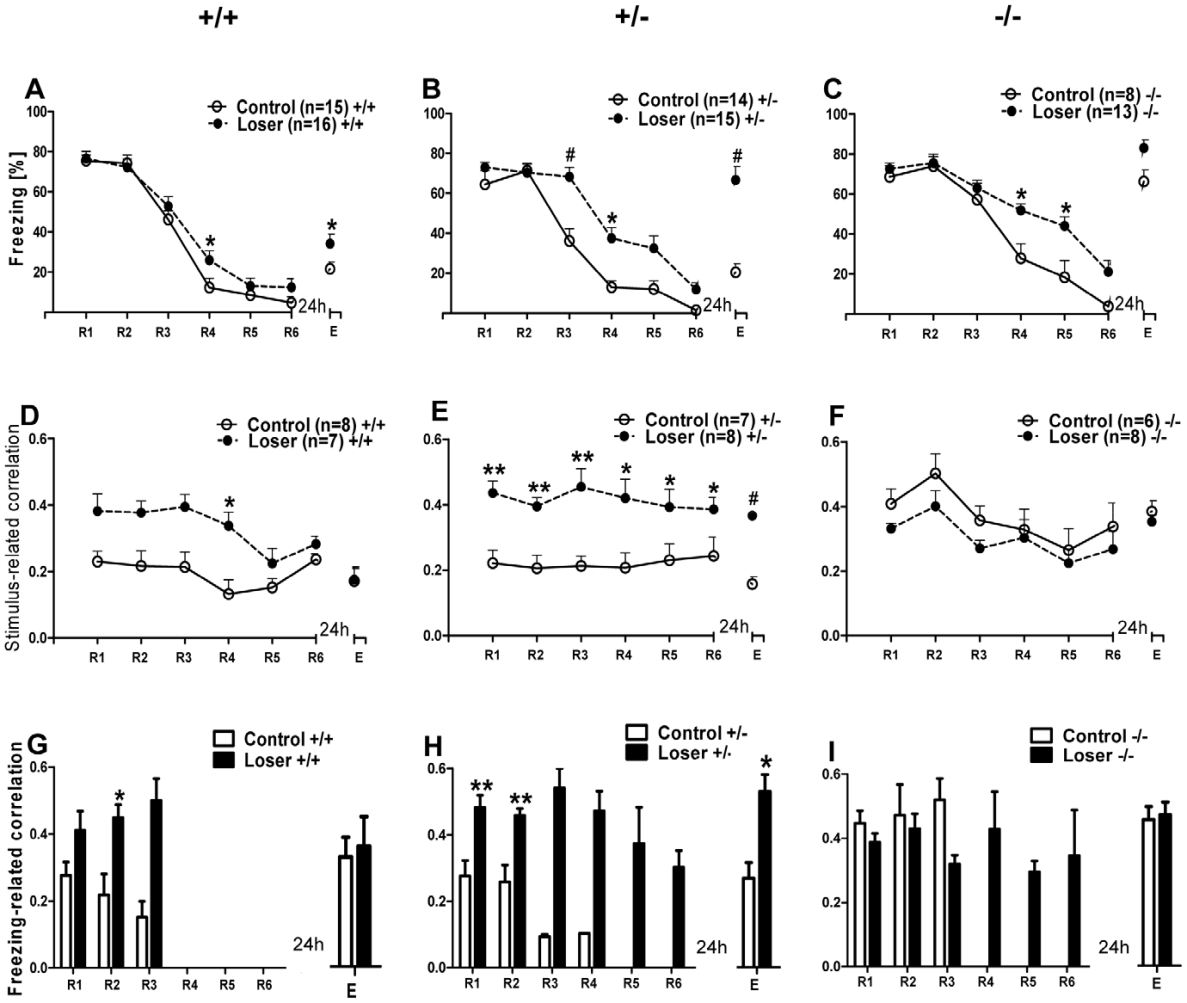
Freezing behavior and theta synchronization in 5-HTT control and loser mice at different stages of fear extinction. **A)** Averaged freezing behavior, **D)** stimulus-related and **G)** freezing-related LA-mPFC theta correlation during the first CS**^+^** across R1-R6 and E in 5-HTT**^+/+^** control and loser mice. 5-HTT**^+/+^** loser mice showed a slightly delayed extinction learning (significant at R4) and impaired recall of extinction (significant at E) accompanied by increased stimulus-related LA-mPFC synchronization during extinction training (significant at R4). Compared to controls, 5-HTT^+/+^ loser mice showed increased freezing-related theta synchronization at early retrieval sessions (significant at R2). **B)** Averaged freezing behavior, **E)** stimulus-related and **H)** freezing-related LA-mPFC theta correlation during the first CS**^+^** across R1-R6 and E in 5-HTT**^+/−^** control and loser mice. 5-HTT**^+/−^** loser mice showed a significantly delayed extinction learning (R3, R4) and impaired recall of extinction (E) accompanied by significant increased stimulus-related LA-mPFC synchronization across extinction learning and recall of extinction. Note, compared to controls, 5-HTT**^+/−^** loser mice showed a highly significant increase of freezing-related theta synchronization across extinction learning (R1-R6) and during recall of extinction (E). **C)** Averaged freezing behavior, **F)** stimulus-related and **I)** freezing-related LA-mPFC theta correlation during the first CS**^+^** across R1-R6 and E in 5-HTT**^−/−^** control and loser mice. 5-HTT**^−/−^** loser mice showed a significant delayed extinction learning (R4, R5). Impaired recall of extinction is indicated by high freezing level in both groups. The stimulus-related correlations in 5-HTT**^−/−^** loser mice were not significantly different from 5-HTT**^−/−^** controls, but both the groups showed high synchronized LA-mPFC activity across R1-R6 and at E. Periods of freezing are accompanied by high freezing-related theta synchronization during extinction learning in 5 HTT**^−/−^** control (R1-R3) and 5 HTT**^−/−^** loser mice (R1-R6) and during extinction recall in both groups. Values are mean ± SEM; *p<0.05, **p<0.01, #p<0.001.

5-HTT^+/+^ loser mice (n = 16) showed an increased level of freezing behavior at R4 and E compared to the control 5-HTT ^+/+^ mice (n = 15; Figure 4A). Repeated measures of one-way ANOVA with group (^+/+^control and ^+/+^loser) as independent variable and session (R1-R6 and E) as dependent variable revealed a significant interaction between the group and session (F (6, 174) = 117.61, p = 0.00001), as well as a main effect of both, session (F (6, 174) = 117.61, p = 0.00001) and group (F (1, 29) = 5.97, p = 0.0209). A post hoc Bonferroni's test revealed that, the 5-HTT^+/+^ losers exhibited delayed extinction and impaired extinction recall compared to wild-type littermate controls, seen in a significantly higher freezing behavior in session R4 and E (p<0.05). Both groups showed a reduction in freezing from R1 to R6 and freezing remained relatively low during E, demonstrating successful extinction of fear (repeated measures one-way ANOVA, F (6, 98) = 81.76, p = 0.00001; post hoc Bonferroni's test in 5-HTT**^+/+^** controls: R1 vs. R3, p<0.0001; R1 vs. R4, p<0.0001; R1 vs. R5, p<0.0001; R1 vs. R6, p<0.0001; R1 vs. E, p<0.05 and repeated measures one-way ANOVA, F (6, 105) = 38.1, p<0.0001; post hoc Bonferroni's test in losers: R1 vs. R3, p<0.001; R1 vs. R4, p<0.0001; R1 vs. R5, p<0.0001; R1 vs. R6, p<0.0001; R1 vs. E, p<0.05).

In the 5-HTT^+/−^ genotype, compared to controls (n = 14), the 5-HTT^+/−^ loser mice (n = 15) showed pronounced freezing behavior during extinction learning, demonstrating a delayed extinction of fear memory. Additionally, 5-HTT^+/−^ loser mice displayed an increased level of freezing behavior during extinction recall compared to 5-HTT^+/−^ controls, demonstrating the disrupted ability to retain the extinction of fear memory (Figure 4B). Repeated measures of one-way ANOVA with group (**^+/−^**control and **^+/−^**loser) as the independent variable and session (R1-R6 and E) as dependent variable revealed a significant interaction between the group and session (F (6, 162) = 5.42, p = 0.00004), as well as a main effect of both session (F (6, 162) = 56.22, p = 0.00001) and group (F (1, 27) = 38.37, p = 0.00001). 5-HTT**^+/−^** losers exhibited significantly higher freezing behavior in R3 (post hoc Bonferroni's test, p<0.0001) and R4 (p<0.05), indicating a delayed extinction compared to controls. Moreover, during extinction recall, the 5-HTT^+/−^ losers displayed significantly increased freezing behavior (Bonferroni's test: E, p<0.001), demonstrating an impaired extinction recall of fear. 5-HTT**^+/−^** control mice showed a reduction in freezing from R1 to R6 and freezing remained relatively low during E, demonstrating successful extinction of fear (repeated measures one-way ANOVA, F (6, 162) = 5.42, p = 0.0004; post hoc Bonferroni's test: R1 vs. R3, p<0.01; R1 vs. R4, p<0.0001; R1 vs. R5, p<0.0001; R1 vs. R6, p<0.0001; R1 vs. E, p<0.0001).

Finally, in 5HTT^−/−^ mice, compared to controls (n = 8), the loser group (n = 13) exhibited delayed extinction of conditioned fear memory, represented by increased levels of freezing behavior during extinction learning. Moreover, during extinction recall, 5-HTT^−/−^ controls as well as 5-HTT^−/−^ losers showed increased level of freezing behavior demonstrating an impaired extinction recall of fear memory (Figure 4C). Repeated measures of one-way ANOVA with group (^−/−^control and ^−/−^loser) as the independent variable and session (R1–R6 and E) as dependent variable revealed a significant interaction between the group and session (F (6, 114) = 2.21, p = 0.047), as well as a main effect of both session (F (6, 114) = 50.37, p = 0.00001) and group (F (1, 19) = 13.01, p<0.0017). Both, the 5-HTT^−/−^ control and loser mice showed a reduction in freezing from R1 to R6, demonstrating a successful extinction of fear (repeated measures of one-way ANOVA, F (6, 114) = 2.21, p = 0.047; post hoc Tukey's test; in controls: R1 vs. R4, p<0.001; R1 vs. R5, p<0.001; R1 vs. R6, p<0.001; in losers: R1 vs. R4, p<0.05; R1 vs. R5, p<0.001; R1 vs. R6, p<0.001). When compared with knock-out littermate (5-HTT^−/−^) controls, the 5-HTT^−/−^ losers exhibited significantly longer freezing time in R4 (post hoc Tukey's test, p<0.05) and R5 (p<0.05), demonstrating a delayed extinction learning. Additionally, 5-HTT**^−/−^** controls as well as 5-HTT**^−/−^** losers showed a significantly impaired recall of extinction (E) (controls: R6 vs. E, p<0.001; losers: R6 vs. E, p<0.001).

In summary, 5-HTT knockout and wild-type mice with repeated social defeat showed impaired extinction learning and extinction recall of fear memory and the effect is more prominent in mice with reduced (^+/−^) or absent (^−/−^) function of the 5-HTT.

### Synchronized theta activity in amygdala-prefrontal cortex network in socially defeated mice (losers) of all three 5-HTT genotypes

Next, synchronized theta activities in socially defeated 5-HTT mice during fear memory extinction and recall were analyzed.

Compared to 5-HTT^+/+^ control mice (n = 8), 5-HTT^+/+^ losers (n = 7) showed highly synchronized theta activities during the presentation of CS^+^ in extinction learning and the correlation values were significantly higher at R4. But in extinction recall, the theta correlations in losers were not significantly different from the control mice (Figure 4D, see also Figure 3B). A repeated measures of one-way ANOVA of LA-mPFC correlation values with group (**^+/+^**control and **^+/+^**loser) as the independent variable and session (R1–R6 and E) as dependent variable revealed a significant interaction between the group and session (F (6, 78) = 2.94, p = 0.012), as well as main effect of both group (F (1, 13) = 11.16, p = 0.0053) and session (F (6, 78) = 6.19, p = 0.000024). When compared with wild-type controls, the 5-HTT**^+/+^** loser mice exhibited significantly higher correlation values at R4 (post hoc Bonferroni's test, p<0.05) and the correlation values remained low during E.

In a next experimental step, correlated activities during freezing episodes were analyzed. Analysis was restricted to sessions R1, R2 and E, because sufficient freezing behavior existed to perform a cross-correlogram. The 5-HTT^+/+^ loser mice showed high freezing-related correlations during extinction, which were significantly higher at R2. However, in extinction recall, theta correlations were not significantly different between loser and control mice (Figure 4G, see also Figure S2). An unpaired t-test revealed that the freezing-related correlation activity in 5-HTT^+/+^ loser mice was significantly higher than in 5-HTT^+/+^ control mice during extinction (significant at R2, p<0.01), but not significantly different between the groups at E.

In 5-HTT^+/−^ mice, compared to controls (n = 7), loser mice (n = 8) exhibited significantly higher stimulus-related correlations during extinction and extinction recall, which demonstrates that the 5-HTT^+/−^ loser mice showed highly synchronized theta activities during fear extinction and recall (Figure 4E). Repeated measures of one-way ANOVA of LA-mPFC correlation values with group (^+/−^control and ^+/−^loser) as the independent variable and session (R1-R6 and E) as dependent variable revealed no significant interaction between the group and session (F (6, 78) = 0.52, p = 0.78921). There was a main effect of group (F (1, 13) = 21.97, p = 0.0004), but not on session (6, 78) = 1.02, p = 0.418). When compared with heterozygous littermate (5-HTT**^+/−^**) controls, the 5-HTT**^+/−^** loser mice exhibited significantly higher stimulus-related correlation values at R1 – R6 and at E (Unpaired t-test, p<0.01; p<0.01; p<0.01; p<0.05; p<0.05; p<0.05 and p<0.0001 respectively). Moreover, compared to 5-HTT^+/−^ control mice, losers exhibited highly synchronized freezing-related correlation with significantly higher values at R1, R2 and E (p<0.01, p<0.01, p<0.05 respectively) (Figure 4H).

In 5-HTT^−/−^ mice, there was no difference in stimulus-related theta activity between the loser (n = 8) and control mice (n = 6). But both the groups showed high LA-mPFC correlation values during extinction and extinction recall, demonstrating that, both the 5-HTT^−/−^ control and loser mice exhibited highly synchronized theta activities during conditioned fear extinction and recall (Figure 4F). Repeated measures of one-way ANOVA of LA-mPFC correlation values with group (**^−/−^** control and **^−/−^** loser) as the independent variable and session (R1–R6 and E) as dependent variable revealed no significant interaction between the group and session (F (6, 72) = 0.35, p = 0.907). There was a main effect of session (F (6, 72) = 7.09, p = 0.00006, but not on group (control and loser) (F (1, 12) = 1.48, p = 0.25). Furthermore, freezing-related correlative activity between control and loser for 5-HTT**^−/−^** mice were not significantly different at R1, R2 and E (p<0.05), but both the groups showed high correlative activities during extinction and extinction recall (Figure 4I).

The cross-correlogram results during the presentation of CS**^−^** in loser and control mice (^+/+^, ^+/−^, ^−/−^), analyzed by one-way ANOVA for repeated measures, revealed no differences between the genotypes during extinction and extinction recall (data not shown).

In summary, impaired fear memory extinction and recall in 5-HTT knockout and wild-type mice with repeated social defeat (losers) is accompanied by highly synchronized theta activities in LA and mPFC.

### Genotype effect on fear memory extinction and theta activity in 5-HTT ^+/+, +/−, −/−^ loser mice

**Figure 5:**
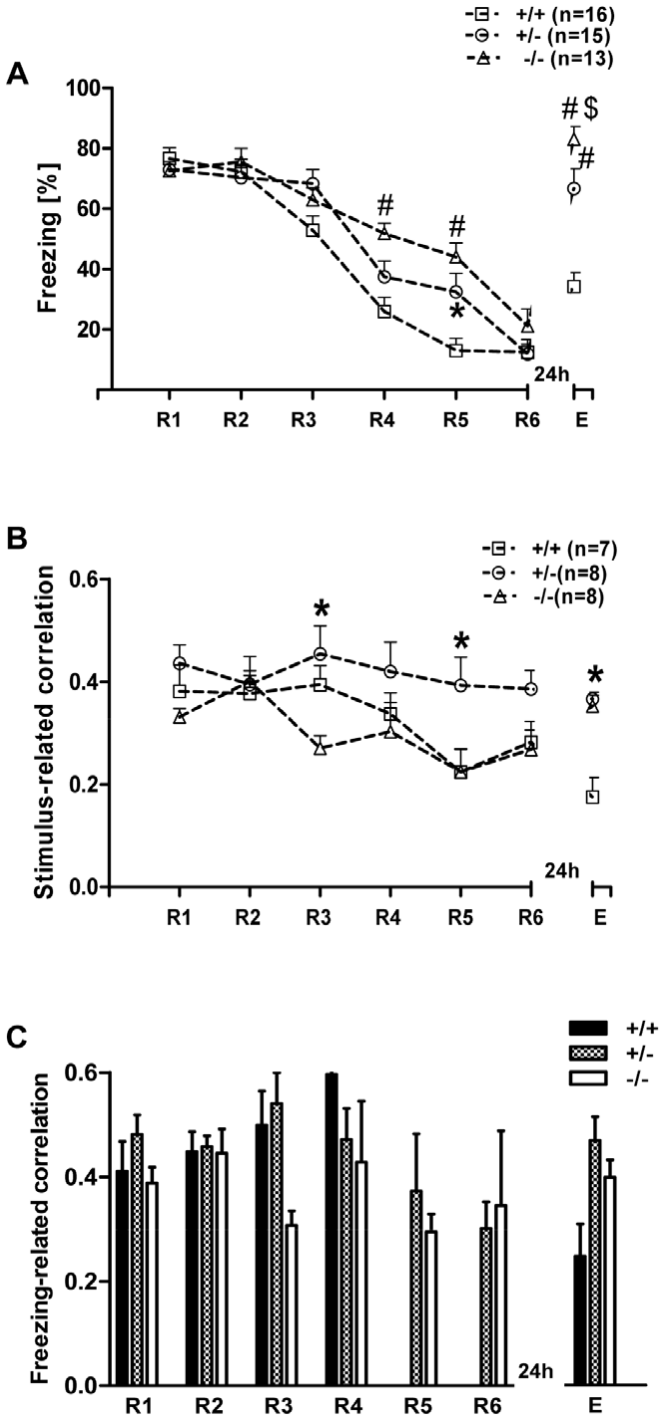
Freezing behavior and theta synchronization in 5-HTT loser mice (all genotypes) at different stages of fear extinction. **A)** Freezing behavior during the first CS**^+^** across R1–R6 and E. Freezing in all three genotypes declined from R1-R6, indicating the standard extinction of fear memory, but both 5-HTT**^+/−^** and **^−/−^** mice showed delayed extinction (in ^−/−^, significant at R4, R5 and in ^+/−^ significant at R5). Twenty four hours later, when all three genotypes were tested for fear extinction recall, 5-HTT^−/−^ mice showed significantly higher freezing behavior (impaired recall) than 5-HTT^+/+^ and ^+/−^ mice, and 5-HTT ^+/−^ were significantly different from 5-HTT^+/+^ loser mice. **B)** Stimulus-related theta synchronization in 5-HTT loser mice at different stages of fear extinction. Compared with 5-HTT^+/+^ (significant at R5) and 5-HTT^−/−^ losers (significant at R3 and R5), the 5-HTT^+/−^ loser mice showed a significantly higher correlation during extinction. 5-HTT **^+/−^** and **^−/−^** loser mice displayed significantly higher correlations at extinction recall (E) compared to 5-HTT^+/+^. **C)** Freezing-related averaged cross-correlation during the first CS**^+^** between LA and IL of mPFC during extinction training (R1-R6) and extinction recall (E) in 5-HTT loser mice. Note high freezing-related theta synchronization in all three genotypes. Values are mean ± SEM; *p<0.05, #p<0.001 compared to 5-HTT^+/+^; $p<0.05 compared to 5-HTT^+/−^.

When comparing the freezing behavior between the three genotypes, 5-HTT^+/−^ and ^−/−^loser mice showed increased freezing behavior during extinction and extinction recall, demonstrating delayed extinction and impaired extinction recall of fear memory compared to 5-HTT^+/+^ loser mice. A repeated measures one-way ANOVA with genotype (**^+/+, +/^** ^−**,** −**/**−^) as the independent variable and session (R1-R6 and E) as dependent variable revealed a significant interaction between the genotype and session (F (12, 246) = 5.16, p<0.0001), as well as a main effect of both genotype (F (2, 246) = 17.73, p<0.0001) and session (F (6, 246) = 81.64, p<0.0001). When compared with 5-HTT**^+/+^** losers, the 5-HTT^−/−^ and 5-HTT^+/−^ loser mice exhibited significantly higher freezing behavior during extinction (in 5-HTT^−/−^ losers, significant at R4 & R5; post hoc Bonferroni's test, p<0.001, p<0.001 respectively; in 5-HTT^+/−^ losers significant at R5; p<0.05). Additionally, the 5-HTT^+/−^ and 5-HTT^−/−^ losers showed significantly higher levels of freezing compared to 5-HTT^+/+^ losers during extinction recall (p<0.001) demonstrating an impaired extinction recall of fear memory (Figure 5A).

During the presentation of the conditioned stimulus (CS^+^), cross-correlograms showed a high level of synchronized theta activities between the LA and mPFC during extinction and extinction recall in losers of all three genotypes (Figure 5B). A repeated measures of one-way ANOVA of LA-mPFC correlation values with genotype (^+/+, +/−,^^−/−^) as the independent variable and session (R1-R6 and E) as dependent variable revealed significant interaction between genotype and session (F (12, 120) = 2.53, p = 0.0053) as well as a main effect of both genotype (F (2, 120) = 4.56, p = 0.0233) and session (F (6, 120) = 5.09, p = 0.0001). When compared with 5-HTT^+/+^ and ^−/−^ loser mice, 5-HTT^+/−^ loser exhibited significantly higher correlation values during extinction learning (^−/−^ vs. ^+/+^ at R3 and ^−/−^ vs. ^+/−^ at R3 and R5; post hoc Bonferroni's test, p<0.05); in extinction recall, both the 5-HTT^+/−^ and ^−/−^ losers showed significantly higher theta correlations compared to 5-HTT^+/+^ losers (p<0.05). Freezing-related correlations between LA and mPFC revealed high values but no significant differences between the loser genotypes (Figure 5C).

In summary, these results indicated a genotype-dependent difference; that is, compared to 5-HTT^+/+^ losers, the 5-HTT^+/−^ and ^−/−^ loser mice showed significantly higher freezing behavior during extinction and extinction recall paralleled with increased theta correlations between LA and mPFC during extinction recall in 5-HTT^+/−^ and during extinction learning and recall in 5-HTT^−/−^ loser mice.

### Directionality of theta synchrony in LA and mPFC

**Figure 6:**
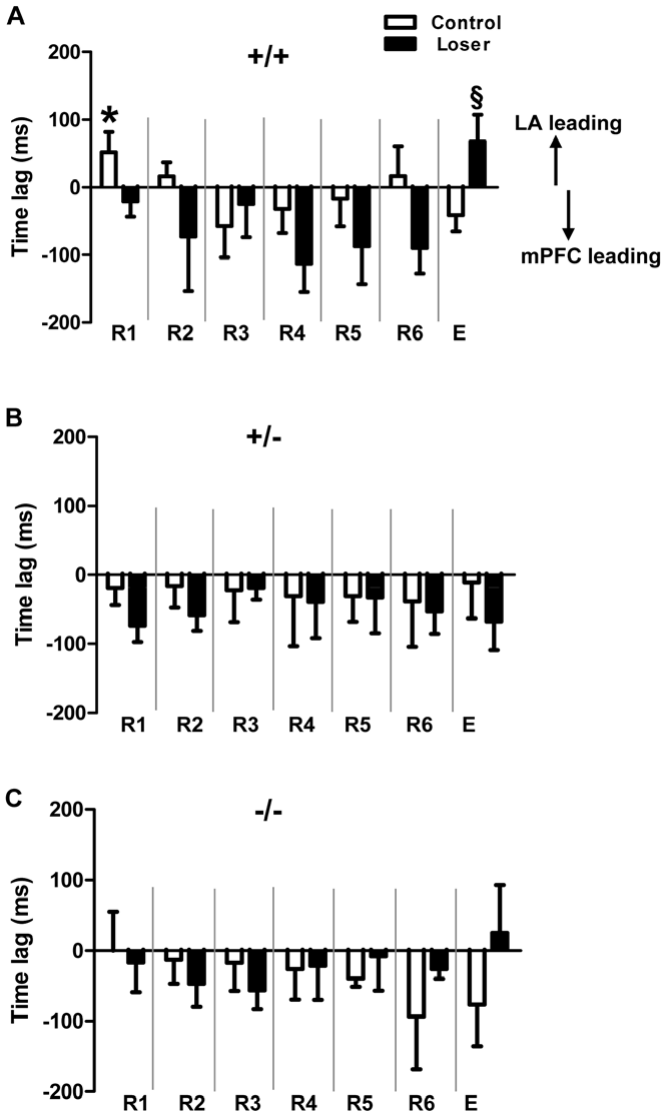
Directionality and time-lag of local field potentials between LA and mPFC at different stages of fear extinction. Negative values indicate mPFC leading, positive values indicate LA leading. **A)** Time-lag between LA and mPFC in 5-HTT^+/+^ naive control and loser mice. In controls, the interaction is started with LA (R1, R2), but later directionality between both is driven by the mPFC (R3, R4, R5 and E), and the amygdala-prefrontal cortex interactions are significantly different between R1 and E (Unpaired t-test, * p<0.05). In 5-HTT^+/+^ loser mice, mPFC is dominating across the entire extinction training, but the directionality changes into LA dominance during E and is significantly different in E between control and loser mice (Unpaired t-test, § p<0.05). **B)** In both 5-HTT ^+/−^ control and loser mice, the mPFC is dominating across R1-R6 and at E. **C)** In both 5-HTT ^−/−^ control and loser mice, the mPFC is leading across R1-R6, but LA is leading in 5-HTT ^−/−^ loser mice at E. Values are mean ± SEM.

In 5-HTT^+/+^ naive control mice (n = 6), during early retrieval (R1, R2), the interactions between LA and mPFC were driven by the LA (positive time-lags), but shifted to mPFC across extinction learning (R3-R5) and rebounded slightly at the end of extinction learning (R6). Interactions driven by the mPFC were most prominent during recall of extinction (E). By comparison, the 5-HTT^+/+^ loser mice (n = 6) showed a strong interaction driven by the mPFC during extinction learning (R1-R6), with a slight shift to amygdala activity during extinction recall (E). The time-lag was significantly different between R1 and E (Unpaired t-test, * p<0.05) in naive controls. Additionally, there was a significant difference in E between naive control and loser mice (Unpaired t-test, § p<0.05) (Figure 6A). In 5-HTT^+/−^, both naive control (n = 6) and loser mice (n = 6) showed a strong directionality of mPFC during extinction learning and extinction recall (Figure 6B). Similar results were obtained in 5-HTT^−/−^ naive control (n = 6) and loser mice (n = 7), in which network activities were driven by mPFC during extinction learning (R1-R6), except a slight rebound LA activity during extinction recall (E) in losers (Figure 6C). In summary, these results indicate that the interactions between LA and mPFC were started with LA at early retrieval sessions and then shifted to mPFC during extinction in wild-type controls. In socially defeated and 5-HTT −/− and +/− control mice, the mPFC leads the LA throughout retrieval and extinction, except a slight LA rebound at E in 5-HTT−/− losers.

## Discussion

The present study presents three major findings: (i) genetically driven loss of serotonin transporter (5-HTT) function produces significant deficits in the ability to retain the extinction of fear memory, concomitant with a change in related neurophysiological (field potential) activities in the LA and IL of mPFC; toward high theta synchrony. (ii) Fear related behaviors and neural amygdala-prefrontal activities can be modulated significantly by negative social experiences in adulthood of mice with deficient (-/-) and reduced (+/-) 5-HTT function. (iii) The directionality between LA and mPFC can be altered by the 5-HTT gene function and social defeat.

The serotonergic system is critically implicated in the pathophysiology and therapeutic alleviation of stress-related disorders such as anxiety and depression. In this system, the 5-HTT is well known as the target of action of serotonin reuptake inhibiters, agents in the treatment of many neuropsychiatric disorders (e.g. PTSD, depression, anxiety disorders, etc.). As already mentioned in the introduction, increased anxiety-like behavior in 5-HTT knock-out mice has interesting parallels with emerging data in humans.

The potential influence of a genetic background in conjunction with environmental factors is a remarkable issue in behavioral phenotyping of mutant mice. Basically, serotonin transporter knock-out mice (5-HTT KO) demonstrate a range of behavioral abnormalities that resemble symptoms of anxiety disorders, like increased fear- and anxiety-related behaviors and reduced exploratory locomotion in behavioral standard tests [1], [33], [51]–[53]; mice with an overexpression of the 5-HTT gene display the opposite phenotype; i.e. reduced anxiety-like behavior [33], [54].

In this study, focusing on fear conditioned and extinction behavior, naive 5-HTT heterozygous (+/-) and homozygous KO mice (-/-) exhibited no deficits in fear acquisition and showed the same level of a fear conditioned memory response (freezing) during retrieval (R1) and extinction learning (R2-R6) compared to wild-type littermates (+/+). The fear memory was extinguished by repeatedly presenting the fear-conditioned stimulus (CS+) in the absence of the aversive stimulus, and all three genotypes of 5-HTT naive mice exhibited a similar progressive decline in conditioned freezing during extinction learning. In contrast, when animals were tested for recall of the extinction of fear memory 24 hours later, homozygous KO mice showed significantly higher levels of freezing, indicating a deficit in the ability to retain extinction of fear memory, which is in line with a previous report [30]. Such a deficit in the ability to acquire or retain extinction memory is a cardinal feature of anxiety disorders such as PTSD [55].

Anxiety-related circuits, involving amygdala, prefrontal cortex and hippocampus are vulnerable to environmental influences [37], [38]. Much data implicate critical interactions between lateral amygdala (LA) and medial prefrontal cortex (mPFC) in the regulation of affective behaviors; as evident when stressful situations lead to increased fear responses [56] or when fear memories are extinguished [40], presumably by 5-HT in the basolateral area which is thought to modulate incoming projections from the prefrontal cortex [57]. In humans, recent investigations focus on amygdala and the PFC as the crucial neural structures involved in the control of stress and fear responses (for review see [58]). In functional MRI (fMRI) studies it was reported that carriers of the 5-HTT s allele exhibited elevated amygdala reactivity [50], [59]–[61]. Later, an fMRI study revealed an increased functional coupling between the amygdala and the ventromedial prefrontal cortex in association with the number of s alleles [50]. In our study, supporting these findings, synchronized field potential activity (theta synchronization at ∼4–8 Hz) between LA and mPFC was increased in 5-HTT knock-out mice; indicating a modulatory effect of loss of 5-HTT function and diminished 5-HT clearance. Such synchronized oscillatory network activities are considered key elements of expression of behavior [42], [62]. In an initial report [46], we demonstrated the integration of such rhythmic activities in form of theta synchronization between LA and the CA1 subfield of hippocampus during the retrieval of fear memory. The theta rhythm is seen in much of the temporal lobe during emotional arousal [63] and theta oscillations have been suggested as a signaling mechanism in amygdala-hippocampal pathways for consolidation of emotional memories [46], [64]–[67]. In 5-HTT +/+ mice of this study, the decline in conditional freezing responses during extinction learning and recall was accompanied by reduced theta synchrony in LA-mPFC as it has been shown in C57BL/6 mice ([47], [49]). Additionally, loss of 5-HTT gene function influenced normal activity in the LA and IL of mPFC in the theta frequency range and interaction between both regions. The serotonergic system, particularly the dorsal raphe nucleus, extensively innervates corticolimbic structures involved in the control of stress and anxiety-related processes such as amygdala, bed nucleus of stria terminalis, mPFC and hippocampus [68]–[70]. There is evidence that the activity of dorsal raphe 5-HT neurons is regulated by the mPFC through the pre- and postsynaptic 5-HT1A receptors; e.g. the application of the 5-HT1A agonist 8-OH-DPAT in mPFC significantly inhibited the firing rate of 5-HT dorsal raphe neurons [71]. In 5-HTT knock-out mice, elevated levels of 5-HT in mPFC might inhibit the serotonergic neurons in dorsal raphe nuclei, thus the 5-HT transmission to the mPFC and presumably to LA is decreasing (for review see also [72]). Moreover, lesioning or silencing the serotonergic raphe nuclei enhances theta activity, suggesting that serotonin normally suppresses theta oscillations [73]–[76]. Indeed, in our study, high LA-mPFC correlated theta oscillation was paralleled by high conditioned fear behavior in 5-HTT KO mice. 5-HTT KO mice displayed a decline in conditional freezing responses during extinction learning, but showed high freezing during recall accompanied by high LA-mPFC theta correlation. These data support the notion that theta synchronization relates to fear memory in these pathways.

The loss of 5-HT clearance in 5-HTT KO mice [77] produces marked increases in extracellular levels and reduced turnover [29] of 5-HT in several brain regions, including frontal cortex [28], and electrophysiological data indicate that activation of serotonergic input to the mPFC inhibits the majority of pyramidal neurons through the 5-HT1A receptors [78], [79]. Decreased 5-HT1A receptor expression has been demonstrated in 5-HTT null mutant mice [80]-[82]. Since a permanently increased extracellular 5-HT level leads to a desensitization [29] and downregulation of 5-HT1A receptors with the consequence of an “uncontrolled” hyperexitability of PFC neurons, indicated by the leading role of the mPFC in the directionality analysis of this study (see discussion below), this would explain increased field potential correlation between LA and mPFC in 5-HTT KO mice. Additionally, heightened amygdala response of individuals possessing the s allele might also reflect increased neuronal excitability, leading to larger local field potential activity and subsequent increases in the fMRI signal [83], presumably due to partial desensitization of inhibitory 5-HT1A receptors following increased synaptic 5-HT [84]. Taken together, such strengthened amygdala-prefrontal (hyper)-activity might reflect the involvement of desensitized and down-regulated 5-HT1A receptors following loss of 5-HTT functionality and increased synaptic 5-HT, acting on excitatory 5-HT receptor subtypes [85]. Thus, marked changes in 5-HT synthesis and turnover seem to be key processes mediating anxiety-like and fearful behaviors in 5-HTT KO mice [29], [36]. Generally, these alterations and reduction of 5-HT1A receptors in 5-HTT KO mice seem to result from more complex changes during neuronal development. Additionally, it has been shown that GABAergic interneuron activity in the BLA can be suppressed by 5-HT1A receptor activation [86], [87]. It is tempting to speculate that alterations of 5-HT receptor functionality of GABAergic cells, particularly in the amygdala, might also be a reason for changing network activities; e.g. intercalated cells (ITC) surrounding the basolateral complex of amygdala provide feed forward inhibition to the basolateral and central amygdala [88] whereas increasing their excitatory drive results in facilitation of extinction learning and recall [89]. Thus, reduced inhibitory activity would lead to increased amygdala output activity of projection neurons with the consequence of impaired extinction and stronger fear expression (for review see [41], [90]).

Phenotypic anxiety-related consequences seem to critically result from gene-environment interactions, in which individual responses to environmental insults are modulated by the genetic make-up [5], [91]. There is evidence that the serotonergic system can be affected significantly by negative experiences; social defeat in particular (loser experience in this study) can have pronounced effects [39], [92], [93]. In this context, a recent study found that humans carrying the low-expressing polymorphic variant (s allele) of the 5-HTT gene has also been associated with heightened risk for major depression in response to experience of stressful life events [94], [95]. When compared with wild-type littermates, 5-HTT −/− [32] and 5-HTT+/− mice [96] experiencing high levels of stressful events (repeated social defeat) showed significantly increased vulnerability to anxiety-related behaviors, altered limbic neuronal morphology and diminished neuroplasticity [97]. Stressed 5-HTT+/− mice showed significantly lower levels of serotonin turnover than WT littermates, selectively in the frontal cortex, which is a structure that is known to control fear and avoidance responses, and which is implicated in susceptibility to depression [96]. Interestingly, in the present study revealed that social defeat leads to impaired extinction and extinction recall of fear memory in all three genotypes; but the effect was more pronounced in loser mice with reduced (+/-) or loss (-/-) of 5-HTT function. Moreover, the heightened freezing behavior during extinction and extinction recall in defeated 5-HTT knock-out mice was associated with increased theta synchrony between LA and mPFC. In 5-HTT+/+ control mice, interactions between LA and mPFC during early retrieval (at R1 and R2) were directed by the LA, and then shifted to mPFC across extinction learning and in extinction recall (Figures 4A and 6A). Social defeat changes the directionality between LA and mPFC in 5-HTT wild-type mice, indicated by the “permanent” leading role of the mPFC, presumably due to “uncontrolled” hyperexcitability of PFC neurons (see discussion above).

Alterations in LA-mPFC directionality in defeated 5-HTT knock-out mice (+/− and −/−) might be masked by the loss of 5-HTT function. Interestingly, behavioral and electrophysiological effects of negative social experience is also prominent in 5-HTT+/- mice, corresponding to the human polymorphism in the 5-HTT regulatory region [23]. These data give evidence for the modulatory effects of 5-HTT gene activity and social experience (gene X environment interaction) in the amygdala-prefrontal cortex network during retrieval and extinction of cued long-term fear memory.

In conclusion, genetically driven loss of 5-HTT function, including altered 5-HT synthesis and turnover, led to impaired extinction and extinction recall of conditioned fear memory. Notably, 5-HTT knockout and wild-type mice exposed to negative experiences, such as social defeat, displayed impairment of extinction and extinction recall of fear memory. These behavioral deficits seem to reflect altered serotonergic transmission affecting the timing of neuronal firing, the specificity of synaptic plasticity at afferent input systems to the amygdala, and the synchronization at theta frequency of functionally connected neuronal populations in the amygdala and medial prefrontal cortex. Amygdala-prefrontal cortex coupling mediated by increased correlated theta activity might reflect a capacity for regulating emotional states. Therefore, a dysfunction in coordinated amygdala-prefrontal coupling seems to be involved in the pathophysiology of 5-HTT-mediated anxiety disorders. Additionally, such dysfunctional coupling may be also associated with altered limbic neuronal morphology and diminished neuroplasticity as reported recently. Nevertheless, our data suggest that fear circuits retain their plasticity during adulthood and can be shaped by negative social experiences during this phase of life and under a specific genetic predisposition. Additionally, socially defeated wild-types and/or a loss of 5-HTT functionality may modulate the directionality between LA and mPFC towards mPFC leads LA. However, further detailed studies, focused on single neural activity (in vivo and in vitro) in the amygdala-prefrontal network are needed to explain cellular and molecular mechanisms influencing fear and anxiety-like behaviors in 5-HTT deficient mice, particularly seen after social defeat in adulthood.

## Materials and Methods

### Animals

#### Ethics Statement

All experiments were carried out in accordance with the European Committees Directive (86/609/EEC) and approved by the local animal care committee (Bezirksregierung Münster, Approval ID 50.0835.1.0, G 53/2005 and LANUV NRW, Approval ID 8.87-51.04.20.09.334).

The experiments were conducted with 9-12 weeks old male serotonin transporter (5-HTT) knock-out mice (KO: ^−/−^; n = 18, losers: n = 13), heterozygous mice (^+/−^; n = 25, losers: n = 15) and wild-type littermates (WT: ^+/+^; n = 27, losers: n = 16) which were backcrossed into a C57Bl/6J background for >10 generations [25]. Mice were bred in pairs of 5-HTT +/- individuals and were raised in the Department of Behavioural Biology in Münster (Germany) in a temperature and humidity controlled animal facility under a 12 h/12 h light/dark cycle (light on at 8.00 AM.). All animals were housed in transparent standard Macrolon cages type III (38 cm × 22 cm × 15 cm) with sawdust as bedding material (Allspan, Höveler GmbH & Co.KG, Langenfeld, Germany), a paper towel, and food (Altromin 1324, Altromin GmbH, Lage, Germany) and water provided *ad libitum.* After weaning at day 21±1 of age, the mice were kept in groups of two to five same-sex littermates until the age of 71±11 days, when they were separated from their littermates and single housed for the rest of the experiment. One week after separation the males were either subjected to social defeat in a resident-intruder paradigm (losers), or they were only shortly taken out of their cages (controls, naive) on three consecutive days. In the resident-intruder paradigm (for details see [32]), the 5-HTT knockout and wild-type mice were singly placed as intruders into the home cages of singly housed adult NMRI males (obtained from Harlan Winkelmann GmbH (Borchen, Germany), which show a high level of intermale aggression. As male mice defend their territory against intruding males, the NMRI males (as residents) usually initiated a fight. The confrontation was observed by an experimenter blind to 5-HTT genotype and stopped when fighting became too escalated, to prevent the mice from injury. The confrontations lasted 10 min at most. According to the definition of Jansen et al. [32] a mouse was categorized as loser, if it showed at least five loser behavioral patterns in each of the three confrontations. In addition, these patterns had to occur at least twice as frequently as winner behavioral patterns. Either after the last confrontation or on the following day, losers and control mice of all three genotypes were transferred (2 minutes car transport) to the Institute of Physiology 1 in Münster (Germany) for electrophysiological analysis. All animals were adapted for 1 week under the same conditions as described above, before starting the electrode implantation and electrophysiological recordings.

### Electrode implantation

For recording of spontaneous extracellular field potentials in freely behaving mice, stainless steel electrodes were implanted unilaterally (left hemisphere) into the LA and IL of mPFC under stereotaxic control. Under deep Narcoren (50 mg/kg, i.p.) anesthesia, stainless steel electrodes were implanted at the following positions. LA: anteroposterior -1.8 mm, lateral 3.9 mm, from bregma and dorsoventral −2.9 mm from the brain surface and IL: anteroposterior 1.8 mm, lateral 0.2 mm and dorsoventral −2.0 mm [98]. For ground and reference, silver electrodes were implanted epidural close to the midline over the cerebellar (anteroposterior -5.8, lateral 1.0 mm) and nasal region (anteroposterior 3.5, lateral 0.5 mm) of the right hemisphere, respectively. After implantation, the electrode ensemble was fixed using dental cement (Pulpdent-GlassLute, Corporation Watertown, MA; USA) and coupled to a rubber like socket for connection with a 5-channel swivel commutator. A surgical recovery period of at least 4 days was given to all animals before fear conditioning.

### Pavlovian fear conditioning, extinction and extinction recall

Experimentally 5-HTT naive and loser mice (^+/+^, ^+/−^, ^−/−^) were tested for fear conditioning, extinction, and extinction recall. All animals underwent the following fear conditioning protocol: Mice were adapted twice to the fear conditioning apparatus with 6 neutral tones, (CS^−^, 2.5 kHz tone, 85 dB, stimulus duration 10 s). On the next day, fear conditioning was performed through two trials of 3 presented CS^+^ (10 kHz tone, 85 dB, stimulus duration 9 s), each co-terminated with an unconditioned stimulus (scrambled foot shock of 0.4 mA, duration 1 s). Twenty four hours later single animals were transferred to the retrieval environment (novel context), connected to a swivel commutator and habituated over a period of 30 minutes before being exposed to 6 retrieval sessions (R1–R6) for extinction training (inter trial interval 30 min), each consisting of a set of 4 CS^−^ and 4 CS^+^. After 24 hours, recall of extinction was tested, E (each consists of 4 CS^−^ and 4 CS^+^ tones; experimental protocol see Figure 1). Fear related behavior (freezing) and electrophysiological field potential activities from LA and mPFC were simultaneously recorded through a differential amplifier, band pass-filtered at 3 and 100 Hz with a 1 kHz sampling rate, during the retrieval, extinction learning and recall of extinction.

### Histology

After completion of all experiments, positions of the recording electrodes were histologically verified (see Figure S3). Under deep Narcoren (60 mg/kg, i.p.) anesthesia the position of the tip of all recording electrodes were marked by small electrolytic lesions (1 mA anodal current for 3 s). The animals were sacrificed with an overdose of Narcoren (100 mg/kg, i.p.), brains were rapidly removed and fixed in 4% phosphate buffered formaldehyde. Lesions were identified in 40 µ frozen frontal sections, counterstained with Cresyl Violet staining.

### Data analysis

Local field potential waveforms (LFPs) were fed through a differential amplifier (DPA-2F; Science Products), band-pass filtered from 1 to 30 Hz, transformed by an analog-to-digital interface (sampling rate, 1 kHz; CED Power 1401; Cambridge Electronic Design), and stored on a personal computer. LFPs from LA and mPFC were simultaneously recorded during the R1–R6 and E sessions of extinction of cued fear while monitoring behavior. LFPs during the presentation of the first CS were analyzed using Spike2 software package (Spike2 version 5.06, Cambridge Electronics Design Ltd., Cambridge, UK). For the analysis of freezing-related correlations, at least 3 seconds defined freezing episodes during the presentation of the first CS were considered. Cross-correlograms between LA and mPFC (mPFC as reference) during the presentation of CS- and CS+ as well as freezing related correlations during CS- and CS+ presentation were calculated and the Y-value of the 2nd positive peak, corresponding to the theta frequency, was taken to quantify correlation levels between both recording areas (for details see Seidenbecher et al. [46]).

Time–frequency color representations (see Figures S1 and S2) were calculated via wavelet transformation of the time-domain data using 120 Morlet wavelets linearly spaced between 4 and 12 Hz using a customized Matlab routine (MathWorks). The color plots depict the modulus of the complex wavelet coefficients (i.e., oscillation amplitude) in units normalized to the time domain amplitude. Freezing (complete immobilization except for respiratory movements) behavior was analyzed, by an observer blind to genotype, as behavioral parameters of fear memory. The percentage of freezing time spent during the first CS- and CS**^+^** presentation across R1–R6 and E were quantified and taken for behavioral analysis. Color-coded power spectra were calculated using a custom Matlab routine (version 7.8.0.347 R2009a, The Math Works, Natick, USA) computing the continuous wavelet transform based upon complex Morlet wavelets. According to Adhikari et al. [99], a cross-correlation of instantaneous amplitudes of field potential oscillation was performed to determine the position of the correlation peak as an indicator of the directionality (lagging or leading) between two oscillating signals in different brain areas. Briefly, this method comprises four steps: first, LFPs are band-pass filtered; second, the instantaneous amplitude of the filtered signals is calculated; third, these amplitudes are cross-correlated and the lag at which the cross-correlation peak occurs is determined; fourth, the distribution of lags obtained is tested to determine if it differs from zero; a positive value indicates that interactions are driven by one region (in the present study the LA) and negative value indicates that the interaction is driven by the other one (here mPFC, see Figure 6) (for more precise details see [99]). Here, field potential waveforms from LA and mPFC were band-pass filtered between 2.5 and 13 Hz and analyzed for time-lags. The cross-correlation between the instantaneous amplitudes of the LFPs from LA and mPFC were computed over lags ranging from +200 to −200 ms.

Statistical analysis of electrophysiological and behavioral data were performed with repeated measures of one-way ANOVA, Bonferroni post-hoc tests, Tukey's post-hoc test and Student's unpaired t-test for p<0.05.

## Supporting Information

Figure S1 **Theta synchronization in different stages of fear extinction in 5-HTT −/−, +/− and +/+ naive control mice.** Field potential activities from lateral amygdala (LA) and the infralimbic region (IL) of the medial prefrontal cortex (mPFC) were simultaneously recorded during retrieval (R1), extinction (R6) and extinction recall (E) while monitoring the behavior of the animal. The figure shows representative traces of local field potentials (LFPs) for each recording area 10 seconds before and during the presentation of the CS^+^ in (A) 5-HTT^+/+^, (B) ^+/−^ and (C) ^−/−^ mice. The behavior of the mice at the particular time is indicated above the traces (f, freezing; r, risk assessment; e, exploration; g, grooming; q, quiet). Color-coded time-frequency spectrograms (wavelet transforms) of the LFP segments before and during presentation of CS^+^ (white bars) for each recording area displayed. The (a) stimulus- and (b) freezing-related cross-correlations are shown beneath the LFP traces, indicating theta synchronization between LA and PFC. All the three genotypes showed increased freezing behavior (highlighted in red) and high stimulus- and freezing-related cross-correlations between LA and PFC at R1. Note, increased freezing and theta synchrony in 5-HTT ^−/−^ mice at E compared to 5-HTT ^+/+^ and ^+/−^ mice. (**X**, not applicable due to low expression of freezing behavior).
(TIF)

Figure S2 **Theta synchronization in different stages of fear extinction in 5-HTT −/−, +/- and +/+ socially defeated (loser) mice.** Field potential activities from lateral amygdala (LA) and the infralimbic region (IL) of the medial prefrontal cortex (mPFC) were simultaneously recorded during retrieval (R1), extinction (R6) and extinction recall (E) while monitoring the behavior of the animal. The figure shows representative traces of local field potentials (LFPs) for each recording area 10 seconds before and during the presentation of the CS^+^ in a (A) 5-HTT^+/+^, (B) ^+/−^ and (C) ^−/−^ mice. The behavior of the mice at the particular time is indicated above the traces (f, freezing; r, risk assessment; e, exploration; g, grooming; q, quiet). Color-coded time-frequency spectrograms (wavelet transforms) of the LFP segments before and during presentation of CS (white bars) for each recording area displayed. The (a) stimulus- and (b) freezing-related cross-correlations are shown beneath the LFP traces, indicating theta synchronization between LA and PFC. Note, increased freezing behavior (highlighted in red) and theta synchrony between LA and PFC in the 5-HTT ^+/−^ and ^−/−^ loser mice at E compared to the 5-HTT ^+/+^ loser. (**X**, not applicable due to low expression of freezing behavior).
(TIF)

Figure S3 **Verification of field potential recording sites.** A) Schematic representation of electrode locations in the infralimbic region (IL) of the medial prefrontal cortex (mPFC) and lateral amygdala (LA). Black dots mark verified field potential recording sites. B) Representative Nissl stained coronal sections showing electrode positions, indicated by arrows, in IL and LA.
(TIF)
