# Supplementary figures and images for: Social Defeat: Impact on Fear Extinction and Amygdala-Prefrontal Cortical Theta Synchrony in 5-HTT Deficient Mice
Source: PLoS One. 2011 Jul 27;6(7):e22600. doi: 10.1371/journal.pone.0022600 (PMC3144906; doi:10.1371/journal.pone.0022600)

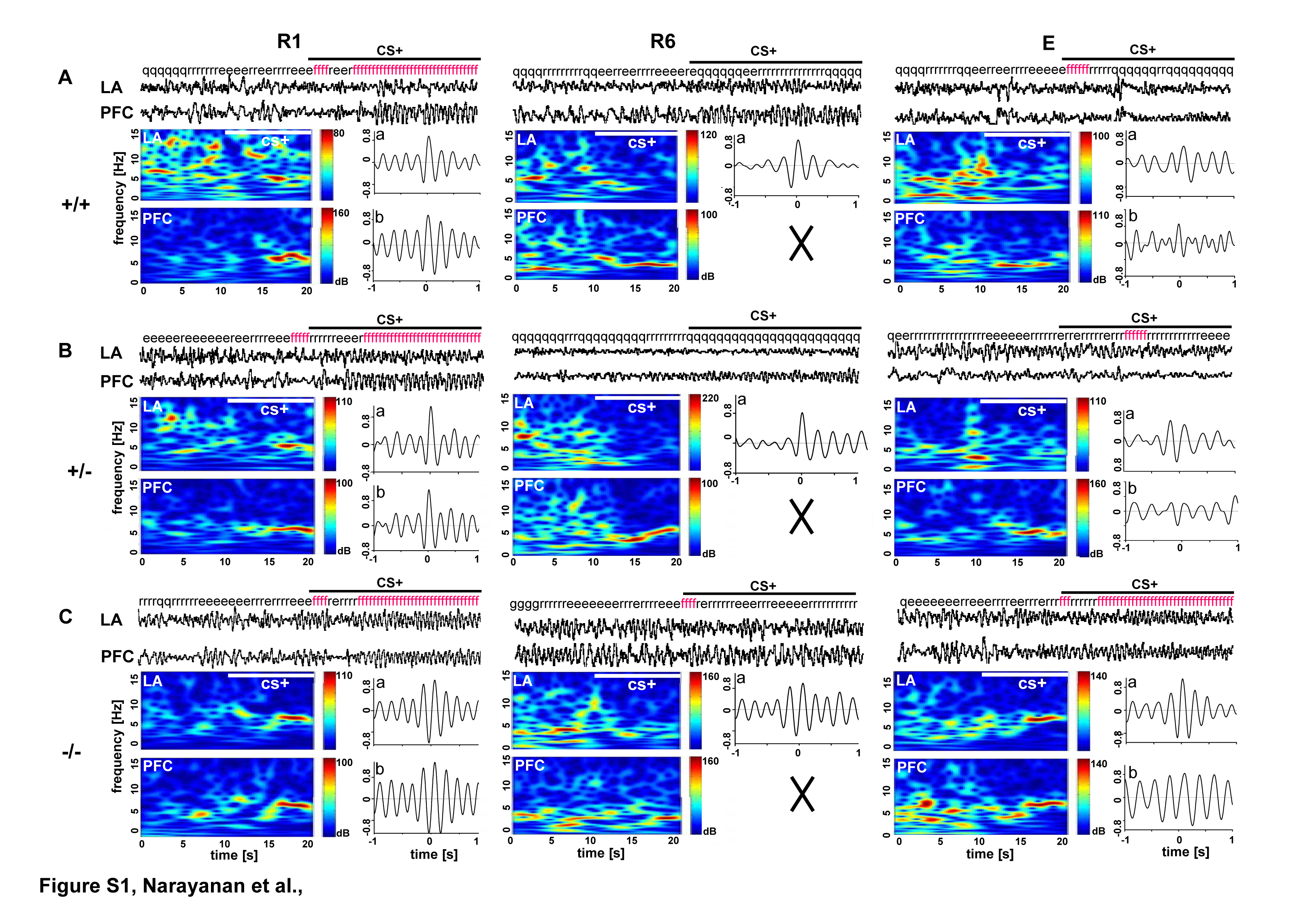

Supplement: Figure S1 — Theta synchronization in different stages of fear extinction in 5-HTT −/−, +/− and +/+ naive control mice. Field potential activities from lateral amygdala (LA) and the infralimbic region (IL) of the medial prefrontal cortex (mPFC) were simultaneously recorded during retrieval (R1), extinction (R6) and extinction recall (E) while monitoring the behavior of the animal. The figure shows representative traces of local field potentials (LFPs) for each recording area 10 seconds before and during the presentation of the CS+ in (A) 5-HTT+/+, (B) +/− and (C) −/− mice. The behavior of the mice at the particular time is indicated above the traces (f, freezing; r, risk assessment; e, exploration; g, grooming; q, quiet). Color-coded time-frequency spectrograms (wavelet transforms) of the LFP segments before and during presentation of CS+ (white bars) for each recording area displayed. The (a) stimulus- and (b) freezing-related cross-correlations are shown beneath the LFP traces, indicating theta synchronization between LA and PFC. All the three genotypes showed increased freezing behavior (highlighted in red) and high stimulus- and freezing-related cross-correlations between LA and PFC at R1. Note, increased freezing and theta synchrony in 5-HTT −/− mice at E compared to 5-HTT +/+ and +/− mice. (X, not applicable due to low expression of freezing behavior). (TIF) [file pone.0022600.s001.tif]

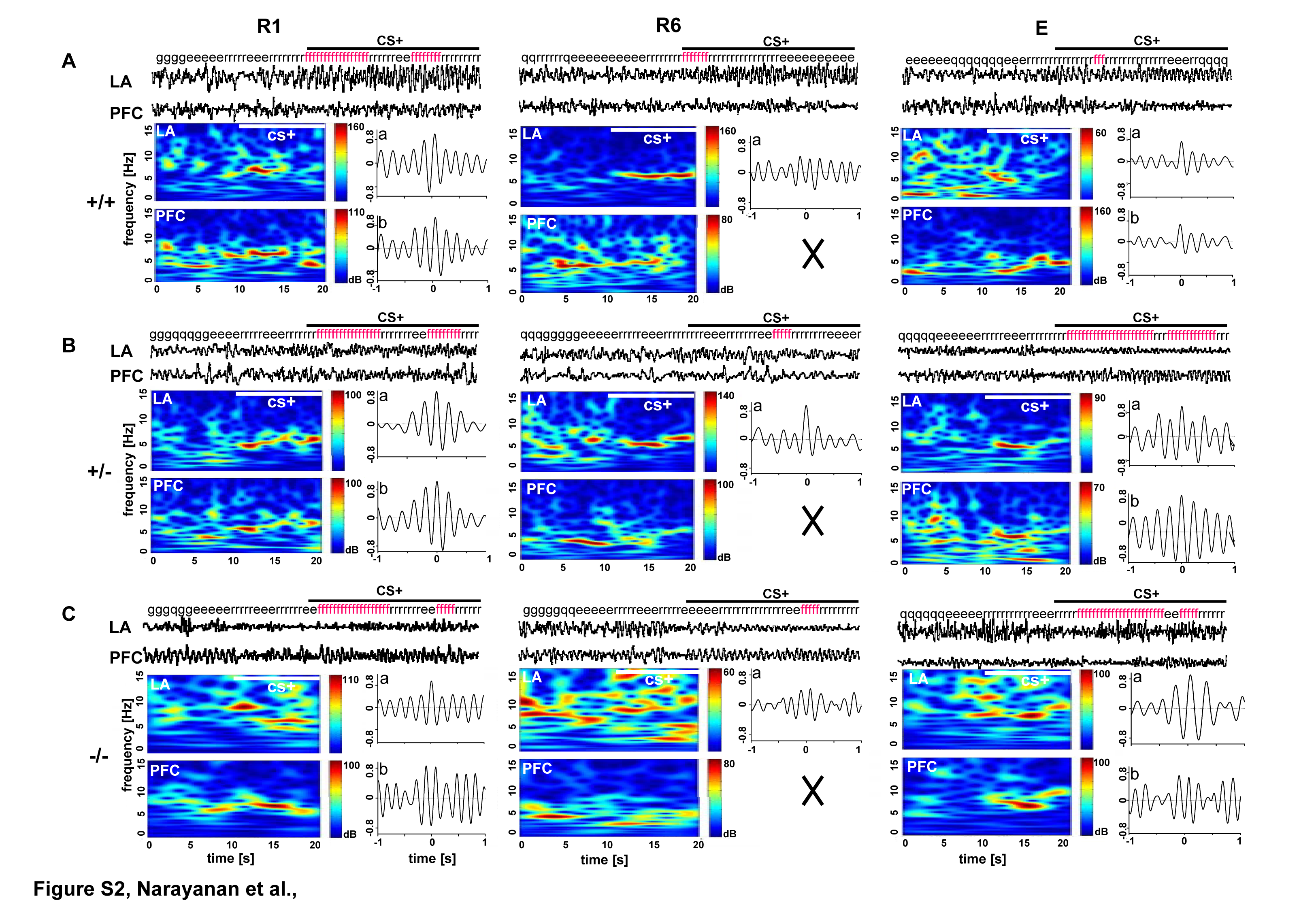

Supplement: Figure S2 — Theta synchronization in different stages of fear extinction in 5-HTT −/−, +/- and +/+ socially defeated (loser) mice. Field potential activities from lateral amygdala (LA) and the infralimbic region (IL) of the medial prefrontal cortex (mPFC) were simultaneously recorded during retrieval (R1), extinction (R6) and extinction recall (E) while monitoring the behavior of the animal. The figure shows representative traces of local field potentials (LFPs) for each recording area 10 seconds before and during the presentation of the CS+ in a (A) 5-HTT+/+, (B) +/− and (C) −/− mice. The behavior of the mice at the particular time is indicated above the traces (f, freezing; r, risk assessment; e, exploration; g, grooming; q, quiet). Color-coded time-frequency spectrograms (wavelet transforms) of the LFP segments before and during presentation of CS (white bars) for each recording area displayed. The (a) stimulus- and (b) freezing-related cross-correlations are shown beneath the LFP traces, indicating theta synchronization between LA and PFC. Note, increased freezing behavior (highlighted in red) and theta synchrony between LA and PFC in the 5-HTT +/− and −/− loser mice at E compared to the 5-HTT +/+ loser. (X, not applicable due to low expression of freezing behavior). (TIF) [file pone.0022600.s002.tif]

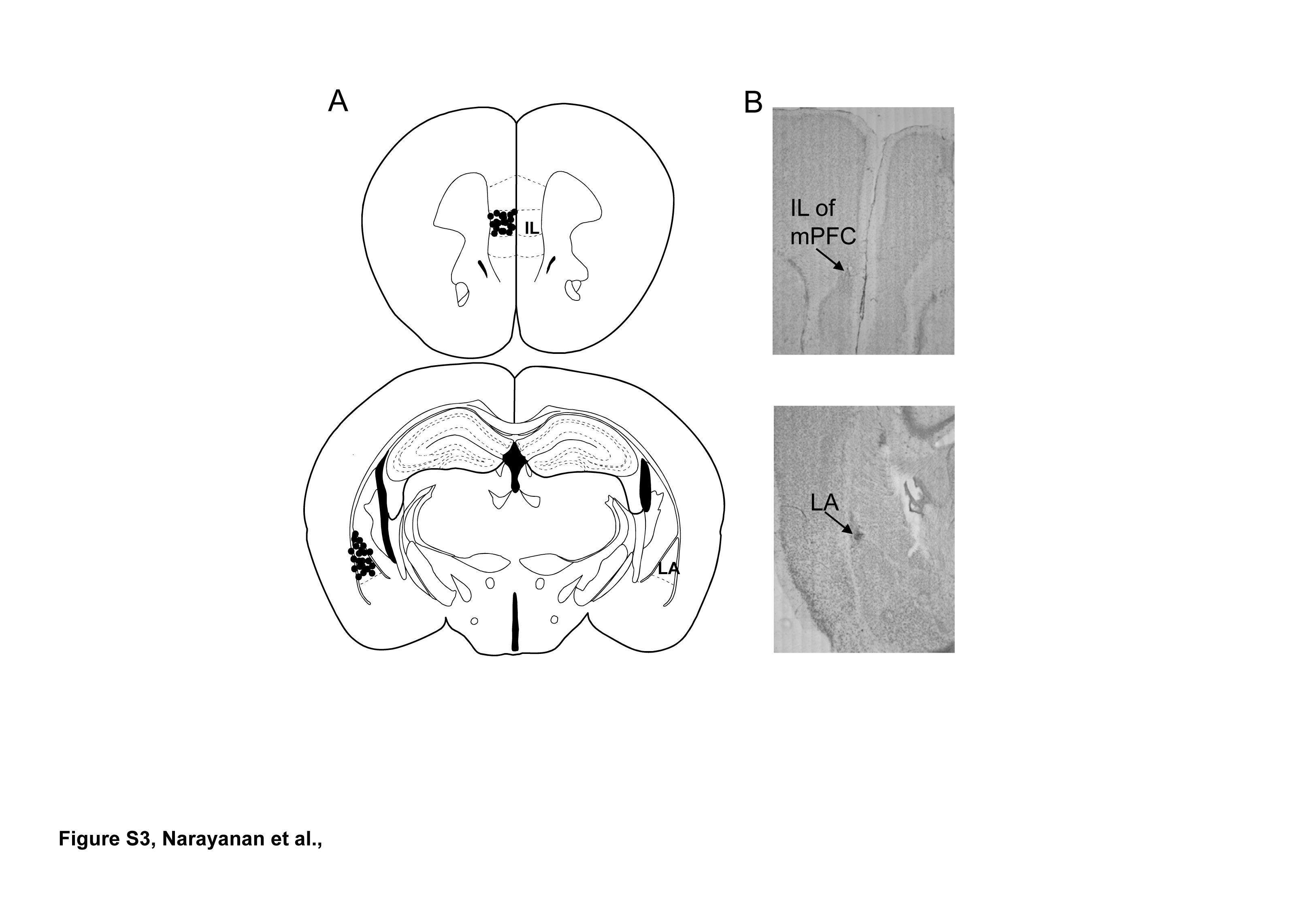

Supplement: Figure S3 — Verification of field potential recording sites. A) Schematic representation of electrode locations in the infralimbic region (IL) of the medial prefrontal cortex (mPFC) and lateral amygdala (LA). Black dots mark verified field potential recording sites. B) Representative Nissl stained coronal sections showing electrode positions, indicated by arrows, in IL and LA. (TIF) [file pone.0022600.s003.tif]
